# Targeting Epigenetic Dysregulation: Antioxidants as Countermeasures Against EDC-Induced Reproductive Toxicity

**DOI:** 10.3390/antiox15060704

**Published:** 2026-06-02

**Authors:** Yue Feng, Dake Chen, Junjing Wu, Xianwen Peng, Shuqi Mei

**Affiliations:** 1 Hubei Key Laboratory of Animal Embryo and Molecular Breeding, Institute of Animal Husbandry and Veterinary, Hubei Academy of Agricultural Sciences, Wuhan 430064, China; fengyue@hbaas.ac.cn (Y.F.); chendake@hbaas.ac.cn (D.C.); wujujing@hbaas.ac.cn (J.W.); pengxianwen@hbaas.ac.cn (X.P.); 2 Hubei Hongshan Laboratory, Wuhan 430070, China

**Keywords:** endocrine-disrupting chemicals (EDCs), reproductive health, epigenetic regulation, oxidative stress, antioxidants, transgenerational

## Abstract

Ubiquitous environmental endocrine-disrupting chemicals (EDCs), including bisphenols, phthalates, and heavy metals, pose a severe and persistent threat to mammalian reproductive health worldwide. Oxidative stress acts as the pivotal mediator which drives epigenetic dysregulation in germ cells upon EDC exposure, including aberrant DNA methylation, abnormal histone post-translational modifications and dysregulated non-coding RNA networks. EDC-induced oxidative stress damages endogenous antioxidant defense systems and inactivates key epigenetic regulators, forming a self-reinforcing cycle of redox imbalance and epigenetic dysregulation, which ultimately leads to impaired gametogenesis, reduced fertility, and transgenerational reproductive abnormalities. This review summarizes current evidence indicating that multiple antioxidants, including melatonin, vitamin C, resveratrol, and epigallocatechin gallate, alleviate EDC-induced reproductive toxicity by targeting epigenetic dysregulation. Their protective effects encompass scavenging excessive reactive oxygen species, activating endogenous antioxidant signaling cascades, restoring activity of epigenetic enzymes, and rectifying aberrant histone modification profiles, contributing to the maintenance of epigenetic homeostasis in germ cells. This review clarifies the intrinsic mechanistic link among EDC exposure, oxidative stress, epigenetic dysregulation and reproductive toxicity, which provides a theoretical basis for formulating reproductive health protection strategies against EDC exposure and guides the exploration of clinical epigenetic biomarkers.

## 1. Introduction

Ubiquitous environmental endocrine-disrupting chemicals (EDCs) are a broad class of exogenous compounds that interfere with the synthesis, metabolism, and action of endogenous hormones in organisms. These compounds include bisphenols, phthalates, heavy metals, polycyclic aromatic hydrocarbons, and cyanobacterial toxins [1,2,3]. With their extensive application in industrial processes, food packaging, and personal care products. Human exposure takes place throughout the lifespan via inhalation, ingestion, and dermal absorption [1]. Epidemiological and experimental studies consistently link EDC exposure to an increased risk of multiple reproductive disorders [4,5,6]. Considering their non-biodegradable nature, bioaccumulative properties, and potential for transgenerational toxicity, EDC-associated reproductive hazards represent a significant global public health concern requiring urgent intervention. To protect public health, regulatory authorities such as the European Food Safety Authority (EFSA) and the U.S. Environmental Protection Agency (EPA) have established permissible exposure limits for major EDCs including Bisphenol A (BPA) and certain phthalates (e.g., DEHP, DBP, BBP) based on comprehensive toxicological risk assessments, providing critical benchmarks for exposure control and food safety evaluation [7,8]. Specifically, following the EFSA 2023 re-evaluation, the tolerable daily intake (TDI) for BPA was set at 0.2 ng/kg bw/day [7]. For phthalates, the TDI values for DEHP, DBP, and BBP are 50 μg/kg bw/day, 10 μg/kg bw/day, and 500 μg/kg bw/day, respectively [8]. Since EDCs typically exist in trace quantities and long-term exposure to them endangers human health, high-precision instruments are required for the detection of food-borne EDCs to ensure reliable quantification, including ultra-high-performance liquid chromatography coupled with tandem mass spectrometry, which offer the sensitivity and specificity required for trace-level quantification of these contaminants [9,10,11].

**Figure 1:**
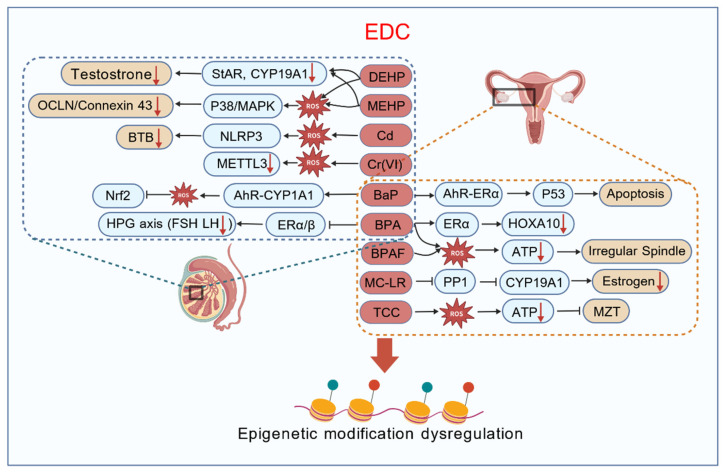
Main pathways through which EDCs affect male and female reproductive capacity. EDCs, including DEHP/MEHP/Cd/Cr(VI)/BaP/BPA, can reduce testosterone production in males, disrupt the structure of the blood–testis barrier (BTB), induce oxidative stress, and cause changes in the hypothalamic–pituitary–gonadal axis, ultimately impairing spermatogenesis. For the female reproductive system, EDCs such as BaP/BPA/BPAF/MC-LR/TCC can exert adverse effects by regulating granulosa cell apoptosis, inducing oocyte spindle abnormalities, reducing estrogen levels, increasing intracellular ROS levels, and inhibiting maternal–zygotic activation. These EDCs not only affect the reproductive system through the aforementioned mechanisms but also via regulating epigenetic modifications. Black arrows indicate promotion, black T-arrows indicate inhibition, and red arrows indicate a decrease. This figure was created with BioGDP.com.

The toxic mechanisms by which EDCs affect the reproductive system have been extensively investigated over the past few decades (Figure 1). Early studies mainly focused on the disruption of nuclear hormone receptor signaling and dysfunction of the hypothalamic–pituitary–gonadal (HPG) axis [12,13]. Emerging evidence demonstrates that oxidative stress represents a key intermediary mechanism underlying EDC-induced reproductive toxicity [14,15]. EDC exposure can promote excessive generation of reactive oxygen species (ROS) in reproductive tissues and germ cells, disrupt endogenous antioxidant defense systems, induce mitochondrial dysfunction, and, subsequently, trigger lipid peroxidation, DNA damage, and germ cell apoptosis [16,17,18]. Advances in environmental epigenetics further suggest that oxidative stress-associated epigenetic dysregulation in germ cells plays a key role in mediating long-term reproductive impairment and transgenerational inheritance of reproductive disorders [19,20]. Unlike genomic mutations, epigenetic modifications, including aberrant DNA methylation, histone post-translational modifications, and dysregulated non-coding RNA networks, are potentially reversible and therefore represent promising targets for intervention, offering new perspectives for preventing and mitigating EDC-induced reproductive toxicity [21].

Within this framework, antioxidants have gained increasing attention in reproductive toxicology due to their excellent capacity to scavenge ROS and modulate endogenous antioxidant defense systems. Recent studies suggest that both natural and synthetic antioxidants can effectively alleviate EDC-induced gamete damage and reproductive dysfunction, with their protective effects increasingly associated with the regulation of epigenetic homeostasis [22,23,24,25]. However, the molecular mechanisms by which antioxidants exert epigenetic protection against EDC-induced reproductive toxicity remain poorly elucidated, and the key scientific issues hindering the clinical translation of antioxidant interventions lack systematic summarization. In particular, a comprehensive review that elucidates the crosstalk between oxidative stress and epigenetic regulation in EDC-induced reproductive toxicity and that examines the epigenetic protective mechanisms of different antioxidants is still lacking.

Therefore, this review summarizes current advances in the field, focusing on: (1) the molecular mechanisms by which EDCs impair gametogenesis and reproductive function via oxidative stress; (2) the epigenetic regulatory networks driven by oxidative stress in germ cells following EDC exposure, including DNA methylation imbalance, abnormal histone modifications, and chromatin remodeling disruption; and (3) the protective effects and specific epigenetic mechanisms of different antioxidants in mitigating EDC-induced reproductive toxicity. This synthesis aims to elucidate the role of redox–epigenetic interactions in EDC-associated reproductive damage, provide a theoretical basis for antioxidant-based intervention strategies to protect reproductive health from EDC exposure, and identify priority directions for future research in this field.

### 1.1. Literature Search Strategy

To retrieve relevant studies investigating the mechanistic associations between EDC exposure, oxidative stress, epigenetic dysregulation, and antioxidant interventions in reproductive toxicity, a systematic literature search was performed. The PubMed databases were searched for original articles and reviews. The search strategy combined keywords related to: (1) environmental toxicants: ‘endocrine-disrupting chemicals’, ‘EDCs’, ‘bisphenols’, ‘phthalates’, and ‘heavy metals’; (2) reproductive toxicity: ‘reproductive toxicity’, ‘gametogenesis’, ‘oocyte maturation’, ‘spermatogenesis’, and ‘fertility’; (3) oxidative stress: ‘reactive oxygen species’, ‘ROS’, ‘oxidative stress’, ‘nuclear factor erythroid 2-related factor 2 (Nrf2)’, and ‘antioxidant defense’; (4) epigenetic regulation: ‘DNA methylation’, ‘histone modification’, ‘non-coding RNA’, and ‘epigenetic reprogramming’; and (5) antioxidants: ‘melatonin’, ‘vitamin C’, ‘resveratrol’, ‘epigallocatechin gallate (EGCG)’, ‘glutathione’, ‘N-acetylcysteine’, and ‘folic acid’. The search was limited to English-language articles. Studies were included if they addressed mechanistic links among EDC exposure, oxidative stress, epigenetic dysregulation, and reproductive toxicity in mammalian germ cells or reproductive tissues, with a focus on antioxidant interventions. Conference abstracts and studies lacking direct relevance to the redox–epigenetic axis were excluded.

### 1.2. Spectrum of EDCs Covered in This Review

This review covers the following classes of endocrine-disrupting compounds and chemical families, including: (1) bisphenols: bisphenol A (BPA) and bisphenol AF (BPAF)—which are widely used in plastic manufacturing and food packaging; (2) phthalates: di-(2-ethylhexyl) phthalate (DEHP), its metabolite mono-(2-ethylhexyl) phthalate (MEHP), and nonylphenol (NP)—commonly found in personal care products and industrial applications; (3) heavy metals: cadmium (Cd), hexavalent chromium (Cr(VI)), methylmercury (MeHg), and lead (Pb)—which are non-biodegradable environmental pollutants with high bioaccumulative potential; (4) polycyclic aromatic hydrocarbons: benzo(a)pyrene (BaP)—a prototypical AhR agonist from combustion sources; (5) cyanobacterial toxins: microcystin-LR (MC-LR)—an emerging EDC-like cyanotoxin; and (6) other emerging EDCs: triclocarban (TCC) and benzophenone-3 (BP-3)—commonly used in personal care products. These compounds were selected based on the following criteria: (a) widespread environmental distribution and well-documented human exposure; (b) well-established evidence of reproductive toxicity in both epidemiological and experimental studies; (c) demonstrated capacity to induce oxidative stress and disrupt endogenous antioxidant defense systems; and (d) reported epigenetic dysregulation, including aberrant DNA methylation, histone modifications, and non-coding RNA expression, in germ cells or reproductive tissues.

## 2. Hormonal Signaling Disruption and Oxidative Stress as Primary Consequences of Environmental Toxicants

EDCs represent a broad class of environmental pollutants that interfere with endocrine system function. They exert adverse effects on reproductive health and embryonic development across multiple species [26]. These compounds disrupt hormonal signaling pathways required for gametogenesis, fertilization, implantation, and fetal development, impairing fundamental reproductive processes [2,27].

### 2.1. Hormone Receptor Dysregulation

One major mechanism through which EDCs impair reproductive function involves mimicking or antagonizing endogenous hormones, thereby disrupting receptor-mediated signaling. Numerous endocrine-disrupting chemicals (EDCs) can interact with nuclear hormone receptors, including estrogen receptors (ERα/β), androgen receptors (ARs), and thyroid hormone receptors (TRs), to exert their effects [28]. This interaction alters the normal expression of downstream target genes in target tissues via epigenetic remodeling. A commonly observed alteration is increased H3K4me3 enrichment in estrogen receptor-positive cells. Certain EDCs, including BPA and vinclozolin, can induce DNA demethylation, suggesting potential modulation of methylation patterns in AR target genes [29]. BPA binds ERα/β and disrupts estrogen-responsive transcription in human granulosa and Leydig cells [30]. This interference disrupts the regulation of the hypothalamic–pituitary–gonadal (HPG) axis, leading to imbalances in follicle-stimulating hormone (FSH) and luteinizing hormone (LH) secretion, ultimately impairing folliculogenesis and spermatogenesis [17,31].

Similarly, phthalates such as di-(2-ethylhexyl) phthalate (DEHP) exhibit anti-androgenic activity, reducing sperm motility and viability and impairing male reproductive function [32,33]. Other environmental toxicants, including microcystins, disrupt receptor signaling via comparable mechanisms. For example, microcystin-LR (MC-LR), an EDC-like cyanotoxin, reduces steroid hormone sensitivity by altering gonadal receptor expression [3,31]. Benzo(a)pyrene (BaP), a polycyclic aromatic hydrocarbon, activates the aryl hydrocarbon receptor and interacts with ERα, leading to histone modification changes in murine germ cells, activation of apoptotic pathways, and reduced fertility in male offspring [34].

### 2.2. Hormone Synthesis and Signaling Dysregulation

Beyond interference at the receptor-level, EDCs perturb endocrine homeostasis by dysregulating enzymatic pathways involved in hormone synthesis, metabolic clearance, and intracellular signaling cascades. Steroidogenic enzymes such as CYP19A1 (aromatase) and steroidogenic acute regulatory protein (StAR) are particularly susceptible to disruption. MC-LR inhibits protein phosphatase 1 (PP1), resulting in reduced CYP19A1 expression and decreased ovarian estrogen synthesis [35]. BPA suppresses murine testosterone biosynthesis by affecting StAR and other steroidogenic enzymes [36,37]. DEHP exerts similar effects by disrupting steroid biosynthesis and reducing testosterone levels. Both BPA and DEHP can disrupt the HPG axis, affecting hormonal balance [4].

EDCs also dysregulate intracellular signaling pathways without binding directly to receptors. BPA activates G protein-coupled estrogen receptor in ovarian granulosa cells, which in turn promotes follicular overactivation and premature follicular atresia [38]. MEHP metabolites induce oxidative stress in Sertoli cells, subsequently activating p38 MAPK signaling pathways. This leads to reduced expression of tight junction proteins such as occludin and connexin-43, resulting in disruption of the blood–testis barrier and impaired spermatogenesis [39]. BPA further alters the activity of homeobox A10 (HOXA10), a key regulator of uterine receptivity, reducing successful embryo implantation rates [40].

### 2.3. Oxidative Stress Dyshomeostasis

Reactive oxygen species (ROS) play a biphasic role in the mammalian reproductive system. At low-to-moderate concentrations, ROS serve as essential signaling molecules that regulate key physiological processes, including oocyte maturation, sperm capacitation, hyperactivation, acrosome reaction, and gamete fusion [41,42]. This physiological redox signaling is tightly controlled by endogenous antioxidant systems. However, either insufficient or excessive ROS levels disrupt reproductive function. ROS deficiency may impair necessary oxidative signaling cascades, leading to reduced fertilization capacity [43]. Conversely, oxidative stress develops when EDCs and other environmental toxicants overwhelm the endogenous antioxidant defense systems, leading to excessive ROS accumulation. This subsequently triggers lipid peroxidation, protein oxidation, DNA damage, germ cell apoptosis, and impaired gametogenesis [44,45]. Therefore, maintaining redox homeostasis, defined as the delicate balance between ROS production and scavenging, is critical for normal reproductive function. Disruption of this balance induced by EDCs underlies much of their reproductive toxicity.

Under physiological conditions, ROS are neutralized by enzymatic and non-enzymatic antioxidant systems, including superoxide dismutase (SOD), catalase (CAT), glutathione peroxidase (GPx), and glutathione (GSH). These defense mechanisms are largely regulated by the Nrf2 signaling pathway. However, EDC exposure frequently suppresses these antioxidant systems. For example, BPA induces excessive ROS generation in germ cells by disrupting mitochondrial electron transport chain function and inhibiting the Nrf2-dependent antioxidant response [36,46]. DEHP promotes oxidative stress and mitochondrial injury in ovarian granulosa cells via the HDAC3-HSP90AA pathway, increasing ROS-mediated damage [16]. BaP activates the aryl hydrocarbon receptor (AHR), which suppresses the Keap1-Nrf2 antioxidant axis, leading to ROS accumulation in murine fetal germ cells [34]. ROS-induced damage further disrupts epigenetic regulatory enzymes, including DNA methyltransferases (DNMTs), ten-eleven translocation enzymes (TETs), and histone-modifying proteins, linking EDC exposure to potential heritable reproductive abnormalities.

Impairment gametogenesis occurs when ROS-mediated processes dysregulate intracellular signaling networks, particularly through interactions between redox imbalance and epigenetic regulation. In spermatogenic cells, EDC-induced oxidative stress impairs sperm function and quality. ROS-mediated lipid peroxidation compromises sperm membrane integrity, resulting in reduced motility and capacitation potential [47,48]. Human studies have reported that BPA exposure is associated with increased seminal malondialdehyde levels and reduced sperm motility [49]. Phthalates such as DEHP and its metabolite MEHP activate ROS-dependent pathways that promote spermatocyte apoptosis and reduce sperm concentration [15]. DEHP also inhibits the Nrf2 pathway and downregulates antioxidant genes, including heme oxygenase-1 (HO-1), exacerbating oxidative stress and activating apoptotic responses [50]. In addition to functional impairment, oxidative stress induces epigenetic changes in sperm. For instance, BaP exposure leads to ROS-associated hypermethylation of imprinted genes and abnormal histone modifications in murine spermatogonia, changes that may be transmitted to offspring [34]. Cadmium (Cd) exposure similarly elevates ROS levels in Sertoli cells, disrupts the blood–testis barrier by downregulating tight junction proteins, and impairs spermatogenesis [51].

In females, EDC-induced oxidative stress disrupts oocyte maturation, spindle organization, and mitochondrial function. BPAF, a structural analog of BPA, induces ROS accumulation in mouse oocytes, leading to DNA double-strand breaks (γ-H2AX), spindle abnormalities characterized by disorganized microtubule arrangement, chromosomal misalignment, and reduced polar body extrusion [52]. Microcystins (MCs), cyanobacterial toxins, increase ROS levels in ovarian granulosa cells, activate p38 MAPK signaling, and induce apoptosis, impairing folliculogenesis and ovulation [53]. Mitochondrial dysfunction represents a key downstream consequence of oxidative stress in oocytes. Multiple EDCs, including triclocarban (TCC) and hexavalent chromium (Cr(VI)), disrupt mitochondrial membrane potential (ΔΨm) and reduce ATP production, impairing cytoplasmic maturation in mice [54]. In murine models, TCC exposure increases ROS levels, induces mitochondrial clustering, and reduces ATP availability, ultimately arresting meiosis and impairing embryonic development [54]. Oxidative stress is consistently associated with reduced ΔΨm, abnormal mitochondrial distribution, and decreased ATP production in oocytes, leading to reduced fertilization capacity and impaired embryonic development, including lower cleavage and blastocyst formation rates [55,56].

These findings indicate that EDCs from structurally diverse classes converge on mitochondrial ROS overproduction and suppression of the Nrf2 antioxidant pathway in both male and female germ cells. The extent of ROS elevation appears to correlate with the severity of epigenetic enzyme dysfunction, including DNA methyltransferase 1 (DNMT1) inhibition by BPA and TET dysregulation by BaP, suggesting a dose-dependent and cell-type-specific redox–epigenetic threshold. However, direct comparative studies quantifying ROS thresholds required to differentially impair DNMT and TET activity remain limited.

### 2.4. Redox–Epigenetic Networks in Reproductive Dysfunction

EDC-induced oxidative stress elicits persistent changes in epigenetic regulation, thereby maintaining reproductive toxic phenotypes. In male murine germ cells, exposure to BaP increases ROS production, which recruits TET enzymes to sites of oxidative DNA damage (8-OHdG). This aberrant targeting promotes the inappropriate oxidation of 5-methylcytosine (5mC) to 5-hydroxymethylcytosine (5hmC), disrupting the methylation patterns of imprinted genes [57]. In female murine germ cells, prenatal exposure to BPA and its analogs induces oxidative DNA damage. Such oxidative stress disrupts the normal process of oogenesis, leading to dysregulated expression of meiosis-related genes and a marked increase in the aneuploidy rate of oocytes [58]. Dysregulation of ROS-associated pathways also extends to early embryonic development. Epigenetic reprogramming errors driven by oxidative stress impair cleavage, blastocyst formation, and implantation, ultimately compromising fetal development. Excessive ROS damages both paternal and maternal genomes and delays active demethylation of the paternal genome. For example, paternal Cd exposure increases murine sperm 8-OHdG levels; upon fertilization, this interferes with paternal genome demethylation, leading to two-cell embryonic arrest [59].

**Figure 2:**
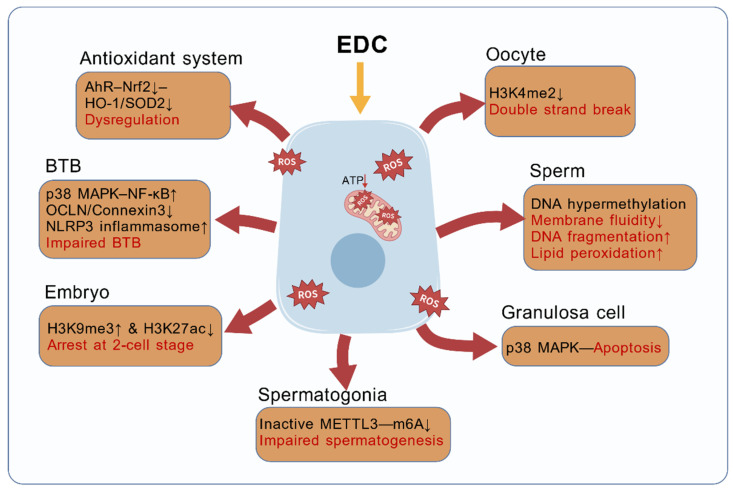
Effects of ROS induced by EDCs on epigenetic modifications in the reproductive system. EDCs enter cells, causing cellular oxidative stress, increased ROS levels in mitochondria, and decreased ATP levels. The elevated ROS levels thereby trigger a series of reactions, such as collapse of the antioxidant system, BTB damage, abnormal histone methylation and acetylation, and abnormal m6A levels. These further lead to embryonic development arrest, spermatogenesis disorders, oocyte double-strand breaks, hypermethylation of DNA in mature sperm, and apoptosis of ovarian granulosa cells. The downward arrows indicate the decreasing levels. The upward arrows indicate the increasing levels. Arrows pointing up and down indicate upregulation and downregulation, respectively. This figure was created with BioGDP.com.

EDCs further disrupt the maternal-to-zygotic transition (MZT), a key developmental window during which embryonic gene expression begins. In murine models, TCC exposure increases ROS-dependent enrichment of repressive histone marks (H3K9me3 and H3K27me3), suppressing zygotic genome activation (ZGA) genes and arresting embryos at the two-cell stage [54]. On the other hand, 3-nitropropionic acid (3-NP) impairs mitochondrial complex II function, leading to ROS accumulation and ATP depletion in ovarian follicles, which further compromises early embryonic development [60,61]. Implantation failure is also closely associated with oxidative stress-mediated epigenetic disruption. BPA downregulates *HOXA10* expression in endometrial epithelial cells, a gene essential for endometrial receptivity [62]. This effect is mediated by decreased promoter methylation and enhanced ERα binding, leading to impaired decidualization and reduced implantation potential. BPA also alters uterine epigenetic landscapes, including histone modifications and non-coding RNA expression profiles [63]. EDCs and environmental toxicants impair reproduction and embryonic development through oxidative stress-driven mechanisms that damage gametes, disrupt embryogenesis, and induce heritable epigenetic alterations (Figure 2). These processes are primarily mediated by suppression of the antioxidant system, mitochondrial dysfunction, and epigenetic reprogramming, with potential for transgenerational transmission. A detailed understanding of these pathways is essential for developing regulatory frameworks and therapeutic strategies aimed at protecting reproductive health.

A key insight from these studies is the bidirectional interaction between ROS and epigenetic regulatory enzymes. ROS not only directly inhibits DNMT and TET activity by oxidative modification of their catalytic domains but also disrupts one-carbon metabolism, reducing the availability of S-adenosylmethionine (SAM). Epigenetic silencing of antioxidant defense genes such as *Nrf2* and *SOD* through promoter hypermethylation establishes a self-perpetuating cycle of oxidative stress. This reciprocal regulation provides a mechanistic basis for the persistence of epigenetic abnormalities following transient EDC exposure.

## 3. Reproductive Epigenetic Dysregulation by Toxicants

### 3.1. DNA Modifications: EDC-Induced Methylation Imbalance in Germ Cells

DNA modifications constitute core epigenetic regulatory mechanisms, including DNA methylation (5mC) and DNA hydroxymethylation (5hmC). DNA methylation is catalyzed by DNMTs and predominantly take place at CpG islands in gene promoters, typically repressing gene transcription. In contrast, DNA hydroxymethylation is produced by TET enzymes and serves as an intermediate in active DNA demethylation. It is typically associated with a more open chromatin state and gene activation. These processes maintain transcriptional stability and genomic integrity [64]. EDCs disrupt the equilibrium of DNA modifications in germ cells by altering the activity of DNMT and TET enzymes, inducing reproductive toxicity. Prenatal exposure to BPA reduces DNA methylation at the Avy metastable epiallele in mouse germ cells. This environmentally susceptible locus is prone epigenetic alterations, and its hypomethylation correlates with altered coat color trats and metabolic disorders in offspring. This hypomethylating effect of BPA is consistent with a potential disruption of DNA methylation maintenance, which could involve interference with DNMT activity or the availability of methyl donors such as SAM [64]. In human male germ cells, combined exposure to DEHP and nonylphenol (NP) results in promoter hypomethylation of multiple genes, including breast cancer 1 (*BRCA1*) and ataxia telangiectasia mutated (*ATM*), leading to altered regulation of DNA repair pathways [21]. Although hypomethylation does not always upregulate transcriptional activity, it increases chromatin accessibility, increasing susceptibility to oxidative DNA damage and contributing to genomic instability in human sperm cells [21].

Cd exposure triggers site-specific changes in DNA methylation. In mouse preimplantation embryos, Cd upregulates histone deacetylase 1 (HDAC1), leading to a significant reduction in methylation at the differentially methylated region (DMR) of the imprinted gene *H19*, while leaving genome-wide repetitive elements, such as long interspersed nuclear element 1 (LINE1), largely unchanged [59]. This locus-specific epigenetic dysregulation results in abnormal H19 expression and impaired embryonic progression from the two-cell to the blastocyst stage [59]. Prenatal exposure to BaP further disrupts DNA methylation dynamics by activating the AHR, which recruits TET2 to the DMRs of imprinted genes such as *H19* and *IGF2* in sperm. This results in an abnormal elevation in 5hmC levels, which can persist in F1 male germ cells and subsequently reduce murine embryo implantation rates in the next generation [34]. Similarly, mice’s in utero exposure to diesel exhaust (DE) has been associated with widespread DNA hypomethylation in neonatal cardiomyocytes, alongside alterations in gene transcription and metabolic function [65]. In *Caenorhabditis elegans*, methylmercury (MeHg) modulates epigenetic regulation of glutathione S-transferase genes through dual mechanisms, including promoter hypomethylation and increased enrichment of H3K4me3, suggesting evolutionary conservation of DNA modification responses to toxic stress [66].

EDC-mediated DNA methylation aberrations are not randomly distributed but preferentially enriched in two major gene categories: imprinted genes (e.g., *H19*, *IGF2*) and genes involved in DNA repair and genomic stability (e.g., *BRCA1, ATM*). This selectivity may reflect differential chromatin accessibility and locus-specific sensitivity to DNMT and TET regulation under oxidative stress conditions. However, this hypothesis requires further validation using high-resolution single-base methylome profiling.

### 3.2. Histone Modifications: EDC-Mediated Dysregulation of Chromatin Activity

Histone modifications are regulated by enzymatic reactions whereby histone methyltransferases, acetyltransferases, and other related enzymes add chemical groups, such as methyl and acetyl groups, to specific amino acid residues on histone tails. Such epigenetic marks modulate chromatin condensation and transcriptional accessibility. For instance, H3K4me3 (trimethylation of lysine 4 on histone H3) is generally associated with transcriptional activation by facilitating access of transcriptional machinery to chromatin. Trimethylation of lysine 9 and lysine 27 on histone H3, respectively (H3K9me3 and H3K27me3), is typically associated with transcriptional repression via chromatin condensation. Endocrine-disrupting chemicals disrupt the balance of histone modifications, altering transcriptional regulation in germ cells. In the human ovarian granulosa cell line COV434, exposure to BP-3 is associated with reduced H3K4me3 levels at DNA repair gene promoters. This effect is driven by the downregulation of mixed lineage leukemia 1 (MLL1), a key histone methyltransferase [21]. Simultaneously, Benzophenone-3 (BP-3) upregulates the histone mRNA degradation factors 5’-3’ Exoribonuclease 1 (XRN1) and decapping mRNA 2 (DCP2), accelerating the degradation of histone H3/H4 mRNAs. These processes reduce chromatin stability and DNA repair capacity, promoting accumulation of DNA double-strand breaks [21]. Prenatal exposure to BaP activates the AhR-ERα signaling axis, leading to increased enrichment of H3K4me3 at the promoter region of the *p53* gene in spermatocytes of F1 male mice. This epigenetic alteration upregulates *p53* expression, activates the Bax-Caspase-3 apoptotic pathway, increases spermatogenic cell apoptosis, and reduces seminiferous tubule epithelial thickness [34].

Histone modification dysregulation also contributes to BPAF-induced reproductive toxicity in mouse oocytes. Exposure to 100 μM BPAF reduces levels of both H3K9me3 (a repressive mark) and H3K27ac (an activating mark). Reduced H3K9me3 levels result in chromatin relaxation, whereas decreased H3K27ac inhibits transcription of meiosis-associated genes including *SYCP3*. These combined changes result in murine oocyte spindle abnormalities, as well as chromosome misalignment [52]. TCC exerts additional epigenetic effects during early embryonic development through bidirectional histone modifications. Following exposure to TCC, levels of both H3K4me3 and H3K9me3 increase, while H3K27ac and H3K9ac decrease. This imbalance between methylation and acetylation inhibits the expression of genes required for murine zygotic genome activation (ZGA), including Zscan4d, resulting in developmental arrest at the two-cell stage [54]. Similarly, heat stress in bovine oocytes has been associated with disturbances in histone modifications and impaired development. Exposure to 41 °C for 12 h reduces H3K4me3 and H3K27me3 levels, which in turn disrupts transcription of mitochondrial-related genes. This includes downregulation of genes such as mitofusin 1 (*MFN1*), leading to reduced mitochondrial function and a decline in oocyte maturation rate in cattle [67]. Histone modification dysregulation represents a central epigenetic mechanism underlying EDC-induced reproductive toxicity. It provides a mechanistic association between environmental exposure, altered chromatin architecture, and impaired germ cell function and reproductive outcomes.

### 3.3. Chromatin Remodeling: EDC-Mediated Dysregulation of Nucleosome Positioning

Chromatin remodeling is an ATP-dependent process executed by chromatin remodeler complexes to reposition and restructure nucleosomes across the genome. This regulation determines the accessibility of promoter regions to transcriptional machinery and other regulatory proteins. When nucleosomes vacate promoter regions, this process facilitates transcription factor recruitment and enhances gene expression, whereas stable nucleosome occupancy limits transcriptional activity [21]. EDCs disrupt the dynamic equilibrium of chromatin architecture in germ cells by interfering with the assembly and function of chromatin remodeling complexes, altering gene regulation during gametogenesis. In *Caenorhabditis elegans*, exposure to DEHP/its metabolite MEHP disrupts ISWI-family chromatin remodeler (SNF2L homolog)-mediated nucleosome repositioning at promoters of DNA repair and meiotic recombination genes, leading to aberrant DSB regulation and germline dysfunction [18]. Similar disruptions in chromatin organization have also been reported in mammalian germ cells [21]. In human ovarian granulosa cells, combined exposure to DEHP and NP disrupts chromatin structure through coordinated downregulation of 573 genes enriched in pathways related to sister chromatid cohesion and centromere positioning. Reduced expression of structural maintenance of chromosomes protein 1A (SMC1A), a key chromatin cohesion factor, contributes to aberrant nucleosome positioning and chromatin instability. These structural alterations have been associated with transgenerational effects, including reduced ovarian reserve in F2 offspring [21]. Similarly, exposure to BP-3 weakens ATM/ATR signaling and reduces the recruitment of SWI/SNF chromatin-remodeling complexes to DNA damage sites. This effect is further exacerbated by decreased H3K4me3 enrichment at promoters of DNA repair genes [21]. Emerging evidence suggests that such chromatin architectural abnormalities may persist beyond the directly exposed generation, indicating that disruption of chromatin remodeling homeostasis may represent a fundamental mechanism underlying EDC-induced reproductive toxicity and its transgenerational inheritance.

## 4. Protective Effects and Mechanisms of Antioxidants on Reproduction via Epigenetic Modulation Against EDCs

EDCs induce reproductive toxicity primarily through oxidative stress and epigenetic disruption, encompassing altered DNA methylation, histone modification patterns, and non-coding RNA profiles. Antioxidants can reduce these harmful effects by restoring redox homeostasis and re-establishing epigenetic regulation. The following sections summarize the mechanisms of key antioxidants in this context.

### 4.1. Melatonin

**Figure 3:**
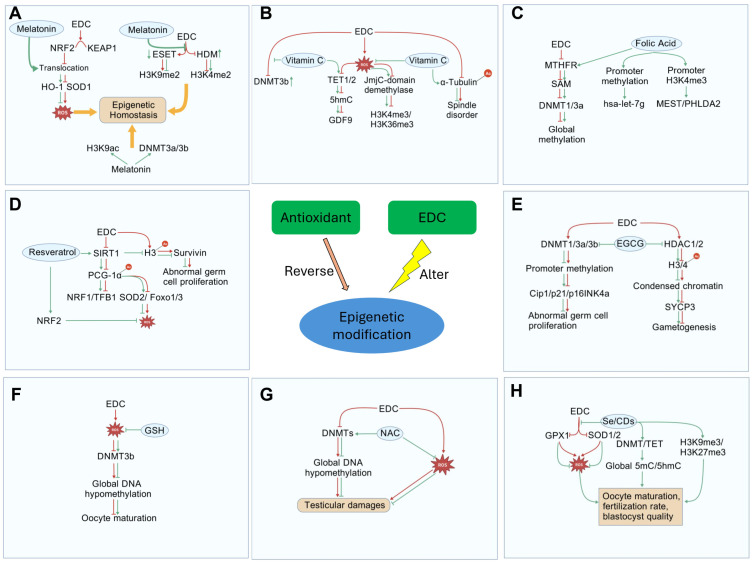
Role of antioxidants in restoring epigenetic modifications disrupted by EDCs. (**A**) Melatonin regulates the nuclear translocation of NRF2 to promote the expression of antioxidant proteins HO-1 and SOD1, inhibits the activity of ESET and activates HDM, and promotes the expression of H3K9ac and DNMT3a/3b, thereby restoring the homeostasis of epigenetic modifications. (**B**) Vitamin C restores normal epigenetic modifications by reducing intracellular ROS levels, upregulating DNMT3b and TETs, and restoring the acetylation of α-tubulin. (**C**). Folic acid restores global methylation levels by promoting the expression of MTHFR protein and supplementing SAM. (**D**) Resveratrol restores the expression of SIRT1, activating the NRF2 pathway; inhibits the acetylation of histone H3; and promotes the acetylation of PGC-1α, ultimately reducing ROS levels and the abnormal proliferation of germ cells. (**E**) EGCG restores promoter methylation levels and histone acetylation levels by inhibiting the expression of DNMT1/3a/3b and HDAC1/2, thereby maintaining normal gametogenesis. (**F**) GSH restores the level of DNMT3b by reducing ROS generated by EDCs, thereby maintaining DNA methylation levels and oocyte maturation. (**G**) NAC maintains DNA methylation levels and repairs testicular damage by restoring the levels of DNMTs and reducing ROS. (**H**) Se/CDs promote the expression of antioxidant proteins to reduce ROS production, while increasing global 5mC/5hmC and histone methylation levels, thereby maintaining oocyte maturation, fertilization rate, and blastocyst quality. Red arrows and T-arrows indicate the major pathways through which EDCs impair reproductive capacity, while green arrows and T-arrows indicate the major pathways through which antioxidants restore the disruptions caused by EDCs. This figure was created with BioGDP.com.

Melatonin confers comprehensive protection against EDC-mediated reproductive injury by coordinately regulating redox balance, mitochondrial function, and epigenetic homeostasis (Figure 3A). One core mechanism lies in activating the Nrf2 antioxidant pathway to mitigate oxidative stress. In copper-exposed porcine oocytes, melatonin enhances Nrf2 activity and decreases ROS accumulation while preventing excessive nuclear translocation associated with pathway overactivation. In the porcine model, this protective effect is stopped in the presence of the Nrf2 inhibitor ML385 [68]. Restoration of redox balance subsequently stabilizes the activity of epigenetic regulators, including histone methyltransferases/demethylases, DNMTs, and TET enzymes, correcting aberrant epigenetic modifications.

Melatonin also normalizes DNA methylation patterns disrupted by environmental toxicants. It reverses age-associated DNA methylation imbalance in porcine oocytes, suggesting its potential to correct EDC-related methylation defects [69]. In pre-pubertal sheep cumulus cells, melatonin reduces methylation at specific CpG sites (e.g., CpG56 and CpG239) within the DNMT3b promoter, leading to upregulated DNMT3b expression and correction of hypomethylation [70]. Moreover, melatonin restores Cd-induced histone modification imbalance and mitochondrial abnormalities in mouse oocytes, an effect likely mediated through redox normalization [71]. It also regulates DNMT3a/3b expression in testes. Under short-day photoperiod conditions in Siberian hamsters, melatonin upregulates *Dnmt3a* in spermatogonia and endometrial tissue, increasing global DNA methylation and supporting photoperiod-dependent reproductive adaptation [72].

Regarding histone modifications, melatonin corrects EDC-induced epigenetic imbalance across multiple models. In Cd-exposed mouse oocytes, it reduces levels of H3K9me2 (a repressive mark) and increases H3K4me2 (an activating mark). These effects are associated with inhibition of the histone methyltransferase ERG-associated protein with SET domain (ESET) and activation of histone demethylase activity [71]. In Cr(VI)-exposed murine spermatogonial stem cells, melatonin downregulates ESET, reduces H3K9me3 levels, and maintains expression of stem cell markers *PLZF* and *GFRα1* [73]. In copper-exposed porcine oocytes, melatonin restores H3K9ac levels through activation of histone acetyltransferases p300/CBP, while also improving mitochondrial membrane potential and ATP production [68]. In low-quality buffalo oocytes, melatonin increases H3K9ac and decreases H3K27me3, promoting expression of oocyte maturation-related genes, including growth differentiation factor 9 (*GDF9)* and bone morphogenetic protein 15 (*BMP15)* [74].

Beyond direct epigenetic regulation, melatonin modulates redox–epigenetic crosstalk to mitigate transgenerational effects of EDC exposure. In mouse oocytes, melatonin may act via nuclear melatonin receptors (NMRs) to help preserve chromatin structure and maintain imprinted gene methylation patterns, potentially buffering against the intergenerational transmission of EDC-induced epigenetic dysregulation [75]. In Cr(VI)-exposed male mice, melatonin reduces germ cell apoptosis (downregulating *Bax* and upregulating *Bcl2*) and restores fertility outcomes by preserving the epigenetic integrity of spermatogonial stem cells [73]. Furthermore, it protects against maternal deficiency-associated offspring metabolic and endocrine disorders by maintaining methylation of circadian and metabolic genes, including those related to insulin sensitivity, limiting oxidative stress-induced epigenetic dysregulation across generations [76].

### 4.2. Vitamins

Vitamins represent a diverse group of essential nutrients with antioxidant and epigenetic regulatory properties. Based on their physicochemical characteristics, vitamins can be classified into hydrophilic (water-soluble) and lipophilic (fat-soluble) compounds [77]. Among these, hydrophilic vitamins have been more extensively studied in the context of EDC-induced reproductive toxicity.

#### 4.2.1. Hydrophilic Vitamins

Vitamin C is a well-characterized water-soluble antioxidant that mitigates EDC-triggered reproductive toxicity via two complementary mechanisms: direct scavenging of ROS and regulation of epigenetic enzyme activity. It acts as a cofactor for TET enzymes involved in active DNA demethylation, as well as for Jumonji C (JmjC)-domain histone demethylases [78]. These enzymes require vitamin C to maintain Fe^2+^-dependent dioxygenase activity. Vitamin C reduces ROS levels, preventing oxidative inactivation of epigenetic regulatory systems [79]. This dual action contributes to the restoration of EDC-induced epigenetic disturbances across reproductive processes (Figure 3B).

In the context of DNA methylation, vitamin C enhances TET-mediated active demethylation. In porcine oocytes exposed to MC-LR, vitamin C restores TET-dependent 5hmC levels at GDF9, a key regulator of oocyte maturation. This restoration improves GDF9 expression and reduces abnormal spindle formation from 48% to 17% [79]. In models of maternal smoking-associated EDC-like oxidative stress, vitamin C enhances TET1 and TET2 activity in placental tissue, correcting aberrant hypomethylation at genes such as protein kinase C alpha (*PRKCA)* and aryl hydrocarbon receptor repressor (*AHRR)*, and supporting placental angiogenesis and fetal lung development [80]. In murine somatic cell nuclear transfer (SCNT) embryos, vitamin C combined with trichostatin A (TSA) enhances TET activity during epigenetic reprogramming, leading to an increase in global 5hmC levels [81]. Vitamin C also stabilizes DNA methylation by preventing EDC-induced suppression of DNMT3b. In ovine oocytes exposed to aflatoxin B1 (AFB1), vitamin C prevents DNMT3b downregulation, limiting global hypomethylation and protecting imprinted gene integrity [82]. In early embryos, it restores impaired paternal genome demethylation following EDC exposure and supports normal blastocyst development [83].

Vitamin C also modulates histone modifications by regulating JmjC-domain demethylase activity under oxidative stress conditions. In MC-LR-exposed porcine oocytes, vitamin C preserves enzymatic function by reducing ROS levels, resulting in restoration of H3K4me3 and H3K36me3, along with preservation of α-tubulin acetylation and spindle integrity [79]. In chicken embryonic gonadal development (E18.5), vitamin C reduces H3K9me3, increases SRY-box transcription factor 9 (*SOX9)* expression, and induces sex-specific gonadal morphological changes, indicating its capacity to modulate epigenetic regulation during reproductive tissue differentiation [84].

Vitamin C supports reproductive epigenetic stability by enhancing TET- and JmjC-dependent demethylation processes while limiting oxidative stress-induced inhibition of epigenetic enzymes. By restoring DNA methylation balance and histone modification patterns, it reduces the persistence of EDC-induced epigenetic abnormalities and may lower the risk of transgenerational transmission of reproductive dysfunction [25].

Folic acid (vitamin B9) is another water-soluble vitamin essential for maintaining reproductive epigenetic homeostasis. Synthetic folic acid exhibits higher bioavailability than natural folates and is therefore widely used in prenatal supplementation to meet increased metabolic demands during pregnancy [85,86]. Folic acid reduces EDC-induced epigenetic disturbances primarily by modulating one-carbon metabolism, ensuring adequate methyl-group availability for SAM synthesis. SAM serves as the principal methyl donor for epigenetic modifications. Through this pathway, folic acid supports DNMT activity and maintains balance with TET-mediated demethylation, restoring disrupted DNA methylation and histone modification patterns associated with EDC exposure (Figure 3C).

Folic acid regulates methylation status at both imprinted and metabolic gene loci. In human studies, folic acid supplementation initiated more than 6 months before conception is associated with increased cord blood leptin (LEP) CpG1 methylation and higher average LEP CpG methylation compared with supplementation initiated later or no supplementation. Continuous supplementation throughout pregnancy also increases average retinoid X receptor alpha (RXRA) CpG methylation [87]. In animal models, dietary protein restriction, which mimics EDC-related metabolic stress, reduces promoter methylation of hepatic glucocorticoid receptor (GR) and peroxisome proliferator-activated receptor alpha (PPARα). Supplementation with 5 mg/kg of folic acid in rat restores these methylation levels to levels comparable to those of controls [88]. Similarly, in folate-deficient conditions associated with neural tube defects, hypomethylation of the hsa-let-7g promoter leads to gene upregulation, whereas folic acid supplementation reverses this epigenetic alteration [89].

Folic acid also modulates histone modification profiles. In vitamin B12-deficient mouse models, high folate intake induces overexpression of placental genes mesoderm-specific transcript homolog (*Mest)* and pleckstrin homology-like domain family a member 2 (*Phlda2)* through increased H3K4me3 enrichment, resulting in an increase for *Mest* and *Phlda2* [86]. These effects counteract repressive epigenetic changes associated with metabolic stress and are dependent on coordinated one-carbon metabolism involving vitamin B12. This interaction prevents the “methyl trap” phenomenon observed under isolated B12 deficiency and helps maintain balanced methyl donor flux, avoiding excessive or dysregulated methylation.

#### 4.2.2. Lipophilic Vitamins

In comparison, lipophilic vitamins including Vitamin E (tocopherols and tocotrienols) and vitamin A derivatives (retinoids) possess well-documented antioxidant properties but have not been systematically evaluated for their epigenetic protective effects against EDC-induced reproductive toxicity. Vitamin E is known to protect against lipid peroxidation in cell membranes and has been shown to modulate gene expression through redox-sensitive transcription factors [90]. Retinoic acid, a major metabolite of vitamin A, regulates gene expression through binding to nuclear retinoic acid receptors (RARs) and retinoid X receptors (RXRs), which can recruit epigenetic co-regulators [91]. However, direct evidence demonstrating that lipophilic vitamins reverse EDC-induced DNA methylation abnormalities, histone modifications, or transgenerational epigenetic inheritance in germ cells remains limited. Future studies should investigate whether these lipophilic vitamins, alone or in combination with hydrophilic vitamins, exert synergistic epigenetic protection against EDC exposure.

### 4.3. Resveratrol

Resveratrol protects against oxidative stress-associated reproductive dysfunction, partially by activating sirtuin 1 (SIRT1), an NAD^+^-dependent deacetylase that regulates histone acetylation and can enhance Nrf2-mediated antioxidant signaling [92]. In oocytes, resveratrol reverses oxidative stress-induced abnormalities in histone acetylation via the SIRT1–PGC-1α pathway, improving meiotic progression and superovulation outcomes in mice [22]. Furthermore, resveratrol enhances antioxidant capacity in reproductive cells by upregulating mitochondrial biogenesis-related genes (*Nrf1*, *Tfb1*) and antioxidant genes (*Sod2*, *Foxo1/3*) through SIRT1-PGC-1α activation, which reduces ROS accumulation and supports oocyte quality following oxidative insult [22] (Figure 3D).

The ability of resveratrol to promote SIRT1-mediated deacetylation of histone H3 and suppress the pro-proliferative gene *Survivin* has been demonstrated in BRCA1-mutant cellular models, highlighting a mechanistic link between SIRT1 activation and epigenetic regulation of cell survival [93]. Emerging evidence suggests that these SIRT1-dependent redox and epigenetic effects may help mitigate aspects of EDC-induced reproductive toxicity, although direct evidence in EDC-exposed reproductive tissues remains limited [22].

### 4.4. Epigallocatechin Gallate (EGCG)

EGCG reverses EDC-triggered epigenetic silencing mainly by direct modulation of epigenetic regulatory enzymes, including DNMTs and histone deacetylases (HDACs), restoring chromatin accessibility and gene transcription. By inhibiting DNMT activity, EGCG reduces promoter hypermethylation and reactivates silenced genes in reproductive tissues, including Cip1/p21 and p16INK4a, restoring transcriptional activity [94]. EGCG suppresses HDAC activity, leading to increased histone acetylation, relaxation of chromatin structure, and improved accessibility of genes required for gametogenesis [94]. In acute promyelocytic leukemia cell models, EGCG induces epigenetic reprogramming through coordinated changes in histone modification patterns. These effects align with its broader capacity to reverse EDC-associated epigenetic dysregulation in reproductive cells [95] (Figure 3E).

### 4.5. Sulfur-Containing Cysteine Derivatives

Glutathione (GSH) and N-acetylcysteine (NAC) are sulfur-containing cysteine derivatives that alleviate EDC-induced epigenetic damage through shared mechanisms involving thiol redox regulation and maintenance of DNMT activity (Figure 3F,G). GSH alleviates EDC-induced epigenetic damage through a dual mechanism involving ROS scavenging and preservation of DNMT function. By maintaining intracellular redox balance, GSH prevents oxidative suppression of DNMT activity and stabilizes DNA methylation patterns in sheep germ cells exposed to toxicants [82]. In ovine oocytes exposed to AFB1, GSH restores intracellular redox status following AFB1-induced depletion, reducing ROS accumulation and maintaining DNMT3b expression. This prevents global DNA hypomethylation and supports methylation of genes essential for oocyte maturation, ultimately reducing developmental abnormalities (Figure 3F) [82].

NAC primarily reverses EDC-induced epigenetic abnormalities by restoring DNMT activity and mitigating oxidative stress-induced testicular damage. BPA exposure is known to inhibit DNMT activity, reduce global DNA methylation, disrupt histone modification patterns, and induce abnormal methylation of imprinted genes, collectively contributing to murine testicular dysfunction and impaired embryonic development [36,96]. NAC restores DNMT expression and enzymatic activity in BPA-exposed male reproductive cells by replenishing intracellular glutathione levels and reducing ROS accumulation, stabilizing DNA methylation homeostasis and protecting germ cell integrity (Figure 3G) [97,98]. Together, these thiol-based antioxidants target the redox–epigenetic axis to restore epigenetic stability in EDC-exposed germ cells.

### 4.6. Selenium-Doped Carbon Quantum Dots (Se/CDs)

Se/CDs represent a novel antioxidant nanoplatform of germ cell quality by simultaneously enhancing endogenous antioxidant defenses and modulating epigenetic profiles. Se/CDs upregulate antioxidant enzymes, including GPX1, reducing ROS levels and promoting a redox environment compatible with epigenetic stability homeostasis in sheep [99]. Se/CDs also regulate DNA methylation by increasing global levels of 5mC and 5hmC, potentially through upregulation of TET expression. Furthermore, they modulate histone modifications by increasing levels of H3K9me3 and H3K27me3, contributing to chromatin stability. These coordinated effects are associated with improved oocyte maturation, higher fertilization rates, and enhanced blastocyst quality (Figure 3H) [99].

### 4.7. Sulforaphane (SFN)

Sulforaphane is a sulfur-containing isothiocyanate derived from glucoraphanin, a precursor abundant in cruciferous vegetables such as broccoli, Brussels sprouts, and cabbage [100]. SFN has emerged as a potent activator of the Nrf2-Keap1 antioxidant signaling pathway. By promoting the nuclear translocation of Nrf2, SFN upregulates the expression of a broad panel of cytoprotective genes, including heme oxygenase-1 (HO-1), NAD(P)H: quinone oxidoreductase 1 (NQO1), and glutathione S-transferases (GSTs), thereby enhancing cellular antioxidant capacity and detoxification of electrophiles and oxidative stress [100,101]. In addition to its antioxidant function, SFN is a well-characterized epigenetic regulator that inhibits histone deacetylase (HDAC) activity. SFN has been shown to inhibit HDAC activity in various cell types, leading to increased histone acetylation (e.g., H3K9ac and H3K18ac) and reactivation of silenced tumor suppressor genes [102]. This dual mechanism, which involves simultaneous activation of Nrf2-dependent antioxidant defenses and inhibition of HDAC-mediated epigenetic repression, positions SFN as a promising candidate for mitigating EDC-induced reproductive toxicity. For instance, SFN attenuates cadmium-induced cytotoxicity and oxidative stress in testicular cells by activating the Nrf2 pathway [103]. Given the shared mechanisms between these toxicants and EDCs, we propose that SFN warrants further investigation as a potential protective agent against EDC-induced reproductive toxicity through its combined antioxidant and epigenetic regulatory activities.

In summary, these antioxidants work together to counteract EDC-induced epigenetic disruption by targeting the oxidative stress epigenetic regulatory axis. They reduce ROS accumulation, restore redox sensitive signaling pathways, such as the Nrf2-Keap1 pathway, and stabilize epigenetic enzyme activity. Through these processes, they correct aberrant DNA methylation patterns and restore histone modification balance. The resulting interplay between redox restoration and epigenetic repair forms an interconnected regulatory network that limits reproductive epigenetic damage induced by EDCs.

## 5. Future Research Perspectives

This review has delineated the core molecular cascade connecting EDC exposure, oxidative stress, epigenetic dysregulation, and reproductive toxicity, and elucidated the epigenetic regulatory mechanisms by which multiple antioxidants mitigate EDC-induced reproductive damage. Despite significant advances in this field, critical knowledge gaps remain, and future research should prioritize the following directions to advance the clinical translation of antioxidant interventions and deepen our understanding of redox–epigenetic crosstalk in reproductive health.

First, single-cell epigenomic profiling is required to unravel cell-type-specific mechanisms underlying antioxidant protection. Current studies mostly assess bulk epigenetic alterations in reproductive tissues, failing to capture the heterogeneous responses of distinct germ cell subpopulations to EDCs and antioxidant interventions. High-resolution single-cell multi-omics approaches will enable the quantification of redox–epigenetic thresholds for epigenetic enzyme inactivation, and the identification of key target loci of antioxidants in specific germ cell types, which is essential to unravel the cell-type-specific protective effects of antioxidants against structurally diverse EDCs.

Second, the transgenerational reversal potential of antioxidant interventions for EDC-induced reproductive pathologies requires systematic assessment. Existing evidence has confirmed that EDCs can trigger heritable epigenetic alterations in germ cells leading to multi-generational reproductive defects, yet few studies have verified whether antioxidants can stably reverse these transgenerational phenotypes and erase the epigenetic memory of EDC exposure across generations. Future studies should define the optimal intervention window, dose–effect relationship, and long-term safety of antioxidants in preventing transgenerational reproductive toxicity, with a focus on their protective effects on imprinted gene methylation homeostasis in germ cells.

Collectively, addressing these key research gaps will not only enrich the theoretical framework of environmental epigenetics and reproductive toxicology, but also provide evidence-based guidance for the development of reproductive health protection strategies against EDC exposure, ultimately reducing the global burden of EDC-induced reproductive disorders and their transgenerational risks.

## 6. Conclusions

EDCs impair reproductive health mainly by disrupting germ cell epigenetic programming via oxidative stress-mediated mechanisms. This review underscores that EDC exposure perturbs DNA methylation, histone modifications, and non-coding RNA regulatory networks, contributing to transgenerational reproductive dysfunction [104]. Oxidative stress emerges as a central mechanistic hub linking environmental exposure to epigenetic dysfunction. It disrupts endogenous antioxidant defense systems, including the Nrf2-Keap1 axis [68], and impairs mitochondrial function, increasing ROS-mediated damage to epigenetic regulatory enzymes.

Antioxidants, including melatonin, vitamin C, resveratrol, and EGCG, counteract these effects through multiple complementary mechanisms. These include scavenging excess ROS to reduce oxidative damage [68,79], activating endogenous antioxidant signaling pathways such as Nrf2 [68,71], and restoring epigenetic stability through regulation of DNA methylation and histone modification systems [69]. Furthermore, some antioxidants modulate TET-mediated demethylation [105] and other epigenetic processes involved in germ cell development [83]. These findings indicate that antioxidants target redox–epigenetic crosstalk to alleviate EDC-induced reproductive toxicity and represent promising therapeutic candidates.

Despite these progresses, multiple critical knowledge gaps still exist. First, systematic comparisons are still required to elucidate how distinct antioxidants differentially modulate specific classes of EDCs, such as bisphenols and phthalates, across diverse germ cell types. Second, limited evidence is available regarding the long-term efficacy of antioxidant interventions in reversing transgenerational phenotypes induced by EDC exposure [104]. Finally, translation to clinical application is limited by insufficient data on human-relevant dosing strategies, tissue-specific delivery systems, and validated epigenetic-recovery biomarkers [69]. Future research should prioritize addressing these limitations through interdisciplinary approaches. Multi-omics integration at single-cell resolution may help delineate EDC–antioxidant–epigenome interactions, while comparative model systems can validate conserved mechanisms of redox–epigenetic crosstalk. Addressing these gaps will strengthen mechanistic understanding and support the development of targeted strategies to protect reproductive health and reduce the transgenerational burden of environmental toxicant exposure.

## Data Availability

No new data were created or analyzed in this study. Data sharing is not applicable to the article.

## Abbreviations

The following abbreviations are used in this manuscript:

3-NP	3-nitropropionic acid
5mC	5-methylcytosine
5hmC	5-hydroxymethylcytosine
AFB1	Aflatoxin B1
AR	Androgen receptor
ATM	Ataxia telangiectasia-mutated
BPAF	Bisphenol AF
BP-3	Benzophenone-3
BRCA1	Breast cancer 1
BTB	Blood–testis barrier
CAT	Catalase
Cd	Cadmium
DMR	Differentially methylated region
DNMTs	DNA methyltransferases
EDCs	Endocrine-disrupting chemicals
EGCG	Epigallocatechin gallate
eNOS	Endothelial nitric oxide synthase
FSH	Follicle-stimulating hormone
GPx	Glutathione peroxidase
GSH	Glutathione
HDACs	Histone deacetylases
HDAC1	Histone deacetylase 1
HPG	Hypothalamic–pituitary–gonadal
LH	Luteinizing hormone
LINE1	Long interspersed nuclear element-1
MCs	Microcystins
MeHg	Methylmercury
MLL1	Mixed lineage leukemia 1
MZT	Maternal-to-zygotic transition
NAC	N-Acetylcysteine
NMR	Nuclear melatonin receptor
NRF2	Nuclear factor erythroid 2-related factor 2
PP1	Protein phosphatase 1
ROS	Reactive oxygen species
SAM	S-adenosylmethionine
SCNT	Somatic cell nuclear transfer
Se/CDs	Selenium-doped carbon quantum dots
SOD	Superoxide dismutase
StAR	Steroidogenic acute regulatory
TCC	Triclocarban
TET	Ten-eleven translocation
TR	Thyroid hormone receptor
TSA	Trichostatin A
ZGA	Zygotic genome activation
